# Analgesic and Anti-Inflammatory Activity of Ambroxol in the Treatment of Endometriosis: An Experimental Study in Wistar Rats

**DOI:** 10.3390/ph19040641

**Published:** 2026-04-18

**Authors:** Gustavo Medeiros Frota, Wilwana Guimarães Barbalho Santos, Joana Tenório-Meireles, Eduardo Rodrigues Silva, Amanda Tissore Forwille Reis, Rennan Abud Pinheiro Santos, Larissa Rodrigues de Sousa, Rafael Antônio Freire Carvalho, Joicy Cortez de Sá Sousa, Eduardo Martins de Sousa, Rafael de Abreu Lima, Rafael Cardoso Carvalho, Marcelo Souza de Andrade, João Batista Santos Garcia, Maria do Socorro de Sousa Cartágenes

**Affiliations:** 1 Experimental Laboratory for the Study of Pain, Federal University of Maranhão, São Luís 65080-805, MA, Brazil; wilwana@hotmail.com (W.G.B.S.); tenoriojoana04@gmail.com (J.T.-M.); eduardo.rs@discente.ufma.br (E.R.S.); amanda.forwille@discente.ufma.br (A.T.F.R.); rennan.santos@discente.ufma.br (R.A.P.S.); larissa.rodrigues1@discente.ufma.br (L.R.d.S.); rafael.afc@ufma.br (R.A.F.C.); joicy.sa@ufma.br (J.C.d.S.S.); rafael.al@ufma.br (R.d.A.L.); carvalho.rafael@ufma.br (R.C.C.); marcelo.andrade@ufma.br (M.S.d.A.); joao.garcia@ufma.br (J.B.S.G.); 2 Laboratory of Immunology and Microbiology of Respiratory Infections, Ceuma University, São Luís 65075-120, MA, Brazil; edmsousa@hotmail.com

**Keywords:** endometriosis, inflammation, pain, ambroxol, rats

## Abstract

**Background/Objective:** This study evaluated the analgesic and anti-inflammatory effects of ambroxol in an experimental model of endometriosis. **Methods:** Ambroxol was administered at doses of 10, 50, and 100 mg/kg (Abx 10, Abx 50, and Abx 100) by daily gavage for 21 days. A medroxyprogesterone-treated group (Progesterone) was included as a positive control. Pain was assessed using validated behavioral tests, including the Rat Grimace Scale (RGS), the von Frey test, and the rotarod test. Additionally, interleukin-1β (IL-1β) levels and total leukocyte counts were measured in peritoneal lavage fluid. The volumetric reduction in endometriotic implants was evaluated by ultrasonography, while histopathological analysis characterized inflammatory infiltrate and epithelial layer integrity using a standardized scoring system. **Results:** All ambroxol doses reduced spontaneous pain manifestations throughout the treatment. The mechanical withdrawal threshold significantly increased from the second week onward, and motor quality improved over the course of the study. A significant reduction in IL-1β levels compared with the negative control (Control(−)) was observed on day 21. Abx 50 and Abx 100 significantly reduced implant volumes (48.2% and 56.2%, respectively) and promoted marked disruption of the endometriotic epithelial layer. When compared with Progesterone, higher doses—particularly 100 mg/kg—demonstrated comparable efficacy. **Conclusions:** Taken together, these pleiotropic effects support the potential for drug repurposing in endometriosis.

## 1. Introduction

Endometriosis is a chronic, estrogen-dependent inflammatory condition characterized by the presence of ectopic endometrial tissue. Affecting approximately 10% of women of reproductive age, it represents a major cause of chronic pelvic pain and infertility. Recent advances in reproductive biology and immunology indicate that the disease is not solely defined by the anatomical presence of lesions. Instead, it involves a profound dysregulation of inflammatory, neuroimmune, and neuroplastic pathways that sustain persistent pain and lesion progression [1,2].

The pathophysiology of endometriosis arises from a complex interplay between immune dysregulation and sustained activation of pro-inflammatory pathways—including NF-κB, JAK/STAT3, and the NLRP3 inflammasome—as well as hormonal and epigenetic alterations that maintain a state of chronic inflammation. Cytokines such as IL-1β, IL-6, and IL-17 play a central role in amplifying these processes, promoting stromal proliferation, angiogenesis, apoptosis resistance, and neural remodeling—processes directly linked to pelvic pain and the dissemination of endometriotic lesions [3,4,5].

Pain associated with endometriosis is multifactorial in origin. It integrates peripheral sensitization mediated by cytokines and neuropeptides; neuroangiogenesis induced by nerve growth factor (NGF) and brain-derived neurotrophic factor (BDNF); neuroimmune responses involving mast cells, macrophages, and T cells; and central sensitization phenomena that perpetuate hyperalgesia. Approximately half of all patients exhibit neuropathic characteristics, reinforcing the need for therapeutic approaches capable of modulating both inflammation and neuronal excitability [6,7].

Currently available therapies, primarily hormonal and surgical, offer only partial and transient relief. They are associated with significant recurrence rates and limited utility for women who wish to conceive or have contraindications to ovarian suppression. This scenario has driven the search for non-hormonal strategies with improved safety profiles that can target both peripheral and central inflammatory and nociceptive pathways [8].

In this context, drug repurposing with established anti-inflammatory and antinociceptive properties emerges as a promising alternative. Among potential candidates, ambroxol, a mucolytic with well-established clinical use, has gained increasing attention due to pharmacological effects extending beyond its traditional respiratory indications.

Experimental evidence indicates that ambroxol exerts analgesic activity by blocking voltage-gated sodium channels, particularly Nav1.7 and Nav1.8, which are associated with neuropathic and inflammatory pain. Furthermore, the drug reduces the release of pro-inflammatory mediators such as IL-1β, IL-6, and TNF-α, attenuates oxidative stress, and modulates glial activation. These findings suggest a multimodal action profile that could be potentially beneficial for chronic pelvic pain associated with endometriosis [9,10].

Despite these promising findings, the potential role of ambroxol in the modulation of pain and inflammatory pathways associated with endometriosis remains poorly explored. Given the significant pain burden associated with this condition and the therapeutic gap for non-hormonal alternatives, investigating whether ambroxol can modulate both inflammatory activity and nociceptive circuits in endometriosis is highly relevant. Therefore, the present study aimed to evaluate the impact of ambroxol on spontaneous pain, mechanical allodynia, motor performance, inflammatory markers, and the integrity of endometriotic lesions in a validated experimental model. Additionally, its efficacy was compared to medroxyprogesterone acetate, a current reference hormonal therapy.

This work provides robust preclinical evidence supporting the potential of ambroxol as a non-hormonal therapeutic alternative for a condition that still lacks safe and effective long-term treatment options.

## 2. Results

### 2.1. Assessment of Spontaneous Pain

**Figure 1:**
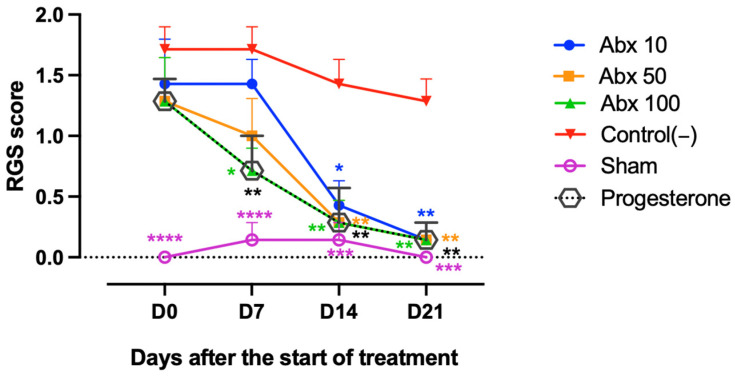
Assessment of spontaneous pain using the RGS. Lines represent the mean ± standard deviation (score range: 0–2). Statistical comparisons were performed between Abx 10, Abx 50, Abx 100, Progesterone, and Sham groups vs. Control(−). Significance levels are indicated as follows: * *p* < 0.05; ** *p* < 0.01; *** *p* < 0.001; **** *p* < 0.0001. Two-way ANOVA with Tukey’s post hoc test. The dashed line represents the baseline (y = 0), offset for visualization purposes.

The Abx 100 and Progesterone groups exhibited similar behavioral profiles throughout the treatment period. During the first week, both groups showed a significant reduction in RGS scores compared with Control(−) (0.714 vs. 1.714; *p* = 0.0017), indicating a relevant analgesic effect. The remaining treated groups did not show a significant reduction in facial pain expression at this stage. By day 14 (D14), a more consistent effect of ambroxol was observed, with convergent reductions in pain scores across all Abx groups (mean values ranging from 0.285 to 0.428) compared with Control(−) (1.428; *p* values ranging from 0.0039 to 0.0177). Similarly, the Progesterone group remained significantly different from the Control(−) group (0.285 vs. 1.428; *p* = 0.0039). By day 21 (D21), scores in all treated groups converged to the lowest value observed (0.142), remaining significantly lower than those of Control(−) (1.285; *p* = 0.0039). No significant differences were observed among ambroxol doses over time (Figure 1).

### 2.2. Assessment of Mechanical Allodynia

**Figure 2:**
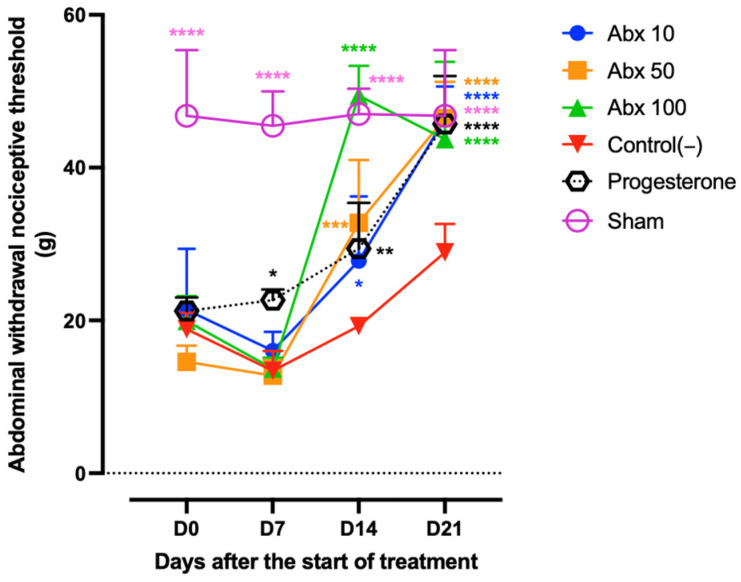
Tactile sensory assessment using the von Frey test. Lines represent the mean ± standard deviation (range: 0.008–300 g of force). Statistical comparisons were performed between Abx 10, Abx 50, Abx 100, Progesterone, and Sham groups vs. Control(−). Significance levels are indicated as follows: * *p* < 0.05; ** *p* < 0.01; *** *p* < 0.001; **** *p* < 0.0001. Two-way ANOVA with Tukey’s post hoc test. The dashed line represents the baseline (y = 0), offset for visualization purposes.

On day 7 (D7), the progesterone group showed a significant increase in mechanical pain threshold compared with Control(−) (22.65 vs. 13.43; *p* = 0.018). Moreover, this group differed significantly from the Abx 50 and Abx 100 groups (22.65 vs. 12.78 and 13.68; *p* = 0.0134 and 0.0231, respectively). Antiallodynic effects of ambroxol became evident on D14, particularly at the 100 mg/kg dose, which reached the highest threshold value (49.42), and differed significantly from Control(−), Abx 10, Abx 50, and Progesterone (*p* < 0.0001). At this time point, the Abx 10 and Abx 50 groups showed increased threshold, approaching the Progesterone group and remaining significantly higher than Control(−) (27.79; 32.79 and 29.39 vs. 19.24; *p* = 0.0492; 0.0001 and 0.0063, respectively). By D21, all treated groups exhibited elevated nociceptive thresholds (ranging from 43.72 to 46.47), reaching values comparable to the Sham group (46.78) and significantly higher than those of the Control(−) (28.91; *p* < 0.0001) (Figure 2). Two-way ANOVA revealed a highly significant time × treatment interaction (*p* < 0.0001), indicating that treatment effects were time-dependent.

### 2.3. Assessment of Motor Activity (Forced Ambulation)

**Figure 3:**
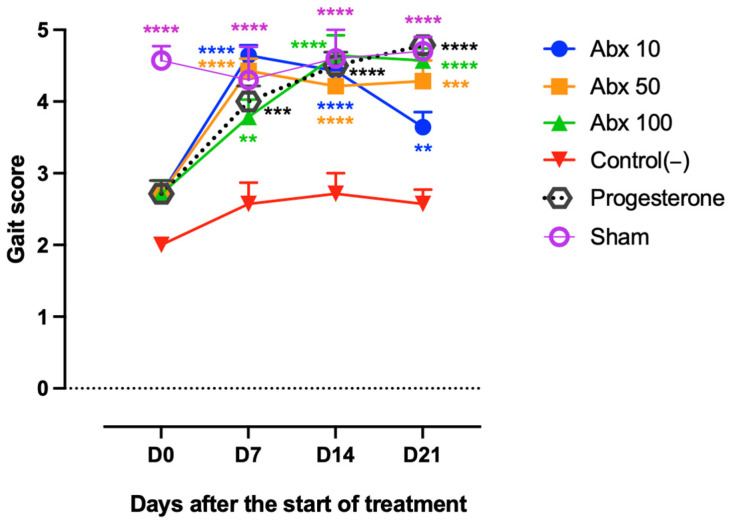
Assessment of motor activity/forced ambulation using the rotarod test. Lines represent the mean ± standard deviation (score range: 1–5). Statistical comparisons were performed between Abx 10, Abx 50, Abx 100, Progesterone, and Sham groups vs. Control(−). Significance levels are indicated as follows: ** *p* < 0.01; *** *p* < 0.001; **** *p* < 0.0001. Two-way ANOVA followed by Tukey’s post hoc test. The dashed line represents the baseline (y = 0), offset for visualization purposes.

Groups subjected to pharmacological treatment exhibited performance comparable to the Sham group, maintaining stable performance and high scores throughout most of the study, indicating preserved motor function with normal or near-normal ambulation. The differences between Abx 10, Abx 50, Abx 100, and Progesterone compared with Control(−) were statistically significant at D7 (*p* < 0.0019), D14 (*p* < 0.0001), and D21 (*p* = 0.0094 for Abx 10; *p* < 0.0001 for the other groups). However, a late decline in motor performance was observed in the Abx 10 group at D21. Although this did not result in a statistically significant reduction compared with Control(−), it was associated with significantly lower performance compared with the Abx 100, Progesterone, and Sham groups (*p* = 0.0378, 0.0044, and 0.0270, respectively) (Figure 3).

### 2.4. Determination of IL-1β Concentration

**Figure 4:**
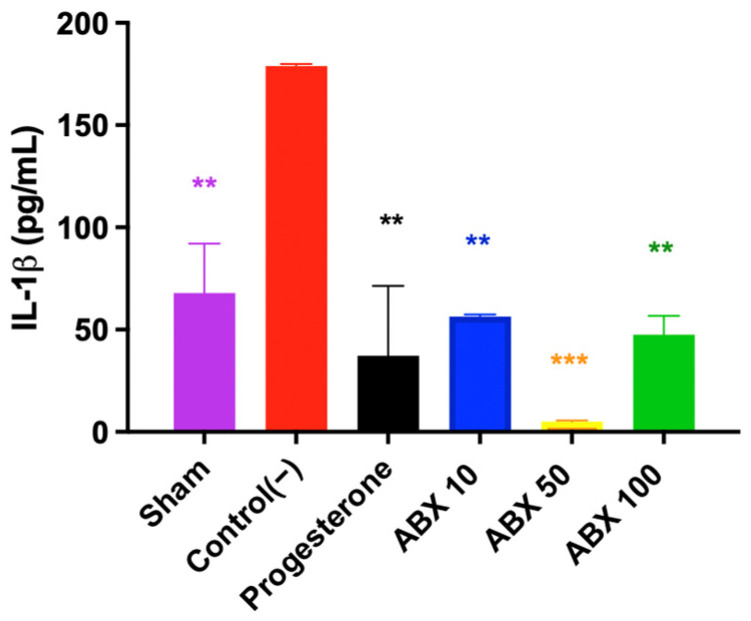
Assessment of IL-1β concentration at D21. Data are presented as mean ± standard deviation. ** *p* < 0.01 for Control(−) vs. Sham, Progesterone, Abx 50, and Abx 100; *** *p* < 0.001 for Control(−) vs. Abx 50 mg. One-way ANOVA followed by Tukey’s post hoc test.

All treated groups (Progesterone and Abx 10, Abx 50, and Abx 100) demonstrated a significant reduction in IL-1β levels compared with Control(−), which exhibited an intense inflammatory response at D21 (mean: 178.96 pg/mL). Pairwise comparisons showed that doses of 10 mg/kg and 100 mg/kg led to statistically significant reductions (56.48 and 47.65 vs. 178.96; *p* = 0.0052 and *p* = 0.0029). Progesterone and Abx 50 also showed strong anti-inflammatory effects (37.23 and 5.01 vs. 178.96; *p* = 0.0015 and 0.0002, respectively), with the latter showing the lowest mean IL-1β levels among the treated groups (Figure 4).

### 2.5. Total Inflammatory Cell Count in Peritoneal Lavage

**Figure 5:**
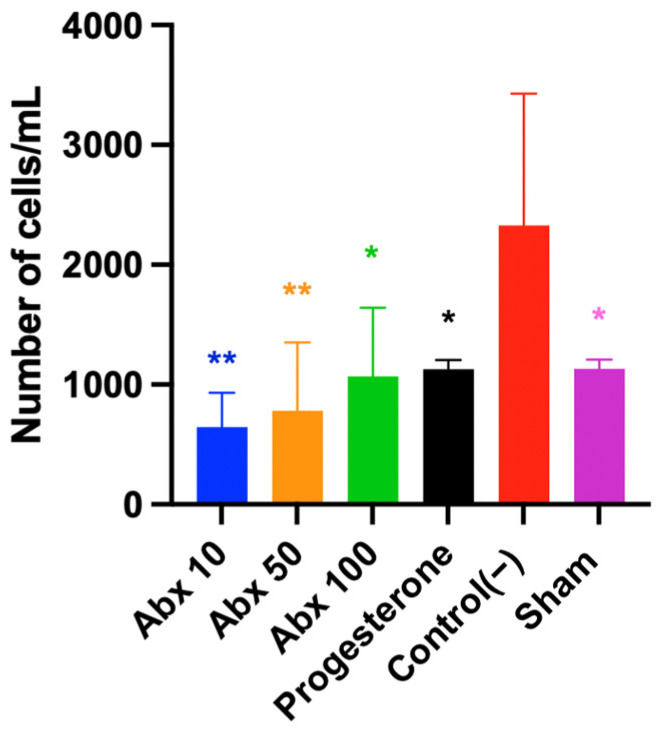
Cell count/mL in peritoneal lavage at D21. Data are presented as mean ± standard deviation. * *p* < 0.05, between Control(−) vs. Abx 100 mg, Progesterone and Sham; ** *p* < 0.01, between Control(−) vs. Abx 10 mg and Abx 50 mg. One-way ANOVA followed by Tukey’s post hoc test.

The Control(−) group exhibited a higher leukocyte count (232,800), indicating intense peritoneal inflammatory activity. Progesterone demonstrated an anti-inflammatory effect, with a cell count similar to the Sham group and significantly lower than that of Control(−) (113,200 vs. 232,800; *p* = 0.03). All ambroxol doses exhibited a response comparable to the progesterone group, with significantly lower cell counts than Control(−) (64,400, 78,000, and 106,720 vs. 232,800; *p* = 0.0013, 0.0032, and 0.0210, respectively), indicating a potent anti-inflammatory effect (Figure 5).

### 2.6. Volumetric Analysis of Implants

**Figure 6:**
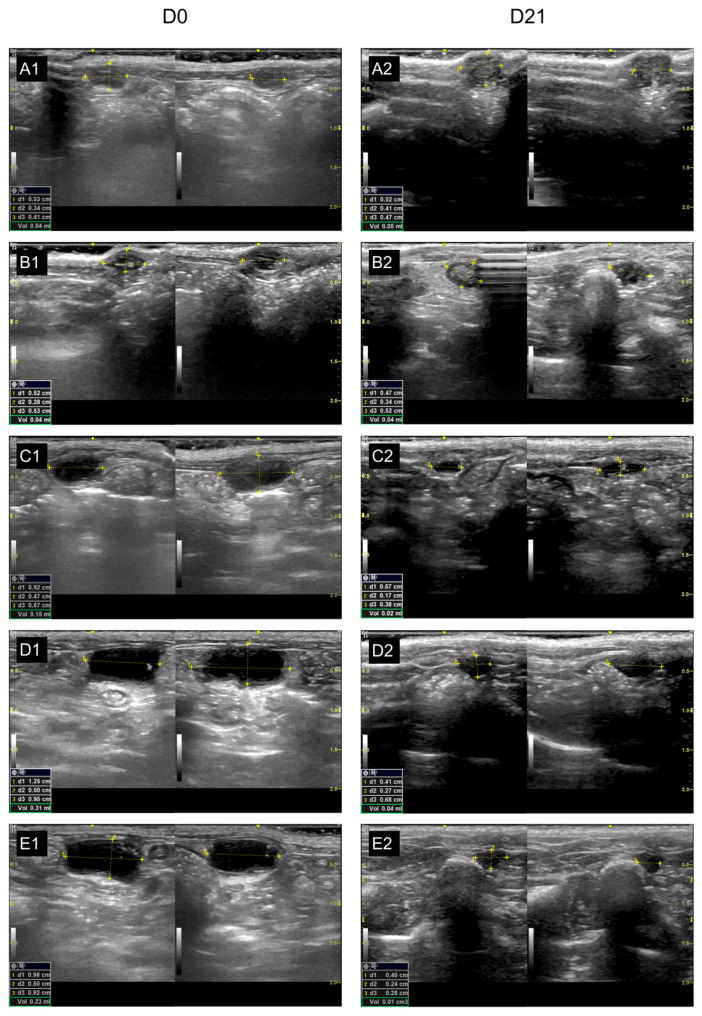
Representative ultrasonographic images illustrating cyst size and volume measurements across experimental groups. Panels (**A1**–**E1**) correspond to D0, and panels (**A2**–**E2**) correspond to D21. Experimental groups are defined as follows: (**A1**,**A2**) Control(−), (**B1**,**B2**) Abx 10, (**C1**,**C2**) Abx 50, (**D1**,**D2**) Abx 100, and (**E1**,**E2**) Progesterone. Cyst dimensions were assessed using three orthogonal diameters (d1, d2, and d3), and cyst volume (Vol) was estimated based on these measurements.

At D21, ultrasonographic analysis (Figure 6) revealed that the Control(−) group maintained endometriotic volumes similar to baseline (47.85 vs. 40). The Progesterone group showed the greatest reduction, both compared with its baseline volume (D0: 45.71 vs. D21: 14.28; *p* < 0.001) and relative to Control(−) at D21 (*p* = 0.0006). In the ambroxol-treated groups, cystic volumes in Abx 50 and Abx 100 decreased significantly between D0 and D21 (*p* = 0.0111 and 0.0006, respectively) and were significantly lower than those of Control(−) at D21 (*p* = 0.0220 and 0.0111, respectively). The Abx 10 group showed a slight volumetric reduction without statistical significance (*p* = 0.0797) (Table 1).

### 2.7. Histological Evaluation

**Figure 7:**
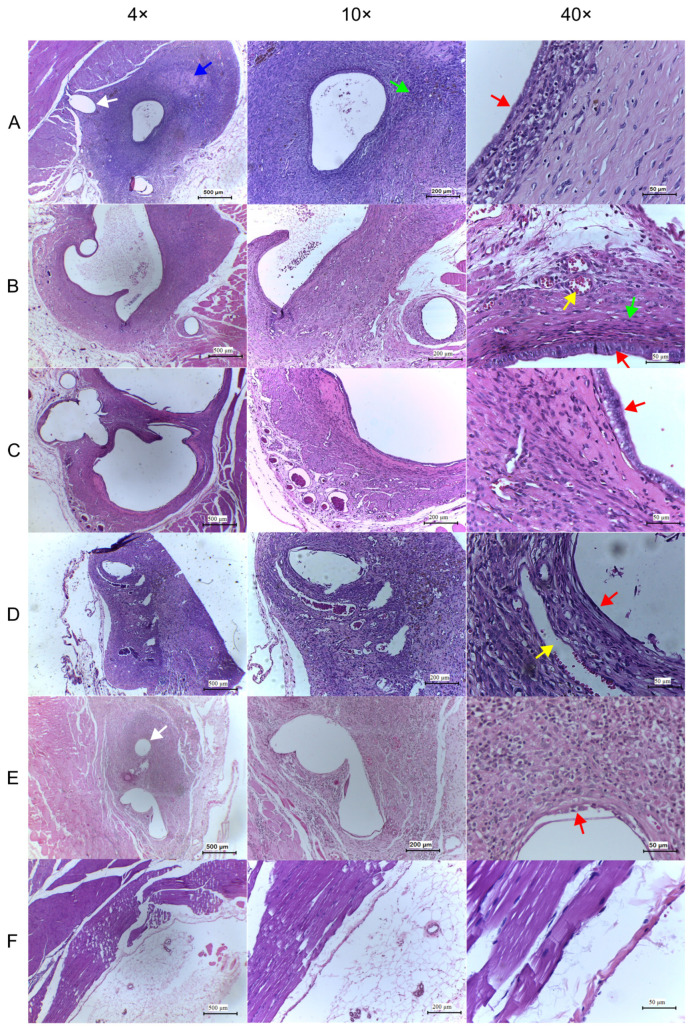
Representative photomicrographs of histological sections of endometriotic implants and internal abdominal wall (Sham group) excised at day 21. Each row corresponds to an experimental group (**A**–**F**), and each column represents a magnification (4×, 10×, 40×). (**A**) Control(−); (**B**) Abx 10; (**C**) Abx 50; (**D**) Abx 100; (**E**) Progesterone; (**F**) Sham. Sections (5 µm) were stained with hematoxylin and eosin (H&E). Colored arrows indicate: red, endometriotic epithelium; blue, fibrosis; green, inflammatory infiltrate; yellow, vascular congestion; white, Nabothian cyst. Scale bars: 500 µm (4×), 200 µm (10×), and 50 µm (40×).

Histological analysis assessed endometrial epithelium, inflammatory infiltrate, stromal cellularity, vascularization, and the extent of implant infiltration into adjacent tissue (Figure 7).

Regarding preservation of the cystic epithelial layer (assessed at D21) using a validated semi-quantitative score, treatment with higher doses of ambroxol (50 and 100 mg/kg) and Progesterone promoted significant reductions in scores compared with Control(−) (1.57, 1.28, and 1.16 vs. 2.83; *p* = 0.0366, 0.0064, and 0.0033, respectively), with no significant differences among these groups. The Abx 10 group (2.57) showed no significant difference compared with Control(−) (*p* = 0.9885) (Table 2).

## 3. Discussion

In the present study, treatment with different doses of ambroxol was associated with a significant reduction in pain and inflammatory activity related to endometriosis. Behavioral assessments demonstrated a progressive reduction in pain expression on the RGS and a substantial attenuation of hyperalgesia from the first week of treatment, as evidenced by improved mobility in the rotarod test. In addition, increased nociceptive thresholds in the von Frey test were observed by the end of the second week. These analgesic effects were sustained until the conclusion of the experiment.

A temporal variation was also observed in the Control(−) group, with a reduction in mechanical sensitivity at later time points. This may reflect intrinsic dynamics of the experimental model, including partial attenuation of inflammation and behavioral adaptation to repeated testing. To account for these time-dependent changes, a two-way ANOVA was applied, enabling evaluation of both time and treatment effects.

Notably, a statistically significant time × treatment interaction (*p* < 0.0001) was identified, indicating that treatment effects were time-dependent and varied throughout the experimental period. Treated groups consistently showed greater and sustained increases in nociceptive thresholds, supporting a true treatment effect.

Therefore, although temporal changes in the Control(−) group may have influenced the interpretation of differences across time points, they do not account for the magnitude or persistence of the effects observed in the treated groups, which reflect a genuine treatment-related response.

Voltage-gated sodium channels are strongly associated with mechanical, thermal, and inflammatory nociception [11]. Patch-clamp studies have demonstrated that ambroxol blocks these channels with 12–21% greater efficacy than classical sodium channel blockers such as lidocaine, benzocaine, and mexiletine [12]. Based on this mechanism, several experimental studies have investigated the role of ambroxol in models of chronic and neuropathic pain.

In chronic pain models, ambroxol (1000 mg/kg) has been reported to produce greater reductions in allodynia and hyperalgesia than gabapentin (100 mg/kg), achieving improvements of up to 70% in inflammatory models such as Freund’s complete adjuvant–induced monoarthritis [13]. Similarly, in spinal cord injury models induced by thoracic compression, oral doses ranging from 30 to 1000 mg/kg significantly reduced mechanical and thermal hypersensitivity without impairing motor performance [14].

These findings suggest that ambroxol exerts analgesic activity predominantly through peripheral blockade of Nav channels, particularly Nav1.8. Consistent with this mechanism, the present study demonstrated effective analgesic activity of orally administered ambroxol in an experimental model of endometriosis-associated chronic pain.

The highest dose tested (100 mg/kg) initially showed the greatest analgesic effect during the first two weeks of treatment, followed by the intermediate dose (50 mg/kg), as observed in the von Frey and RGS assessments. From day 14 onward, however, all treated groups exhibited stable analgesic responses that persisted until the end of the experiment. The absence of statistically significant differences among doses at the final time point suggests that even the lowest dose tested was sufficient to maintain meaningful analgesic activity.

Nevertheless, this observation should be interpreted in light of these limitations. The relatively small number of animals per group may have limited the statistical power to detect subtle dose–response differences. Taken together, the results do not support a strictly dose-dependent effect; rather, they indicate a partially dose- and time-dependent response, with greater effects observed at higher doses in the early phase, likely associated with more pronounced cystic volume reduction and epithelial regression, followed by a convergence of responses at later time points.

Additional experimental evidence supports the analgesic potential of ambroxol in neuropathic pain models. In a murine model of oxaliplatin-induced peripheral neuropathy (10 mg/kg, intraperitoneal), intravenous administration of ambroxol at doses of 12.5 and 37.5 mg/kg significantly reduced cold allodynia in the cold-plate test. In that study, the lower dose required combination with pregabalin to achieve significant analgesia, whereas the higher dose was effective during the early phase of allodynia [15].

In the present study, ambroxol treatment also produced clear anti-inflammatory effects. A reduction in the inflammatory response was evidenced by decreased IL-1β levels at day 21, reduced inflammatory infiltrate within the lesions, and decreased cellularity in the peritoneal lavage. These findings suggest that ambroxol may be associated with the modulation of inflammatory signaling pathways.

One possible anti-inflammatory mechanism underlying these effects involves modulation of the NF-κB/NLRP3/caspase-1/IL-1β inflammasome axis, as suggested by previous studies. Through modulation of these signaling pathways, ambroxol may, at least in part, attenuate the release of pro-inflammatory cytokines and reduce the intensity of the inflammatory cascade associated with endometriosis.

Among the treated groups, the 50 mg/kg dose exhibited the lowest mean IL-1β levels. However, this finding should be interpreted with care. Cytokine concentrations in inflammatory models often display substantial inter-individual variability and do not necessarily follow a strictly linear dose–response pattern. Importantly, all samples were collected, processed, and analyzed under identical experimental conditions, minimizing methodological variability.

Therefore, the markedly low values observed in the Abx 50 group may reflect biological variability rather than a consistent pharmacological superiority of this dose. Notably, all treated groups demonstrated a consistent reduction in inflammatory markers compared with controls, supporting the overall anti-inflammatory effect of ambroxol.

The anti-inflammatory activity of ambroxol has also been reported in other experimental models. In a chronic obstructive pulmonary disease model induced by lipopolysaccharide and cigarette smoke exposure, oral ambroxol significantly reduced serum IL-1β, PGE_2_, and COX-2 levels, while decreasing pulmonary expression of MUC5AC and TLR4 [16].

Similarly, in an acetic acid–induced ulcerative colitis model, ambroxol administration reduced both macroscopic and microscopic tissue damage and restored the colon weight-to-length ratio. These effects were accompanied by increased antioxidant activity (Nrf2, HO-1, and catalase) and reduced levels of inflammatory mediators, including NF-κB, MPO, IL-6, and TNF-α, along with increased IL-10 levels [17].

The histological findings of the present study were consistent with this anti-inflammatory profile. Progressive reductions in inflammatory cell infiltration were observed across treated groups, with stromal cellularity in the Abx 100 group approaching that observed in progesterone-treated animals.

Cytological analysis of the peritoneal lavage similarly demonstrated a significant reduction in total leukocyte counts across all treated groups. These observations reinforce the anti-inflammatory potential of ambroxol in endometriosis-associated inflammatory activity.

Regarding epithelial preservation and volumetric changes in excised endometriotic cysts, antiproliferative and volume-reducing effects were observed only with the intermediate (50 mg/kg) and high (100 mg/kg) doses of ambroxol, as well as with progesterone. These treatments resulted in significant atrophy of both the endometriotic epithelial layer, as reflected by lower Keenan scores [18], and cyst volumes at D21.

Over the past decade, several studies have suggested potential antitumor and antiproliferative effects of ambroxol hydrochloride, with promising results. Preclinical in vitro studies have demonstrated significant effects of ambroxol in lung carcinoma (A549) cell cultures, including increased activation of pro-apoptotic pathways, downregulation of anti-apoptotic pathways, and enhanced cytotoxicity [19,20,21].

In a mouse model bearing subcutaneous xenografts of multiple myeloma, treatment with ambroxol (100 mg/kg, intraperitoneally) for 21 days resulted in a significant reduction in tumor growth (*p* < 0.01) [22].

These current lines of evidence suggest potential tissue-protective and antiproliferative properties of ambroxol, particularly with sustained administration at higher doses, and support its possible role as an adjuvant agent in the control of tumor progression.

The significant reductions in cystic volume and epithelial integrity observed with higher doses of ambroxol appear to play a key role in enhancing analgesic responses in behavioral pain assessments from the early weeks of treatment. The 100 mg/kg dose achieved the highest nociceptive thresholds (von Frey test) and the lowest pain expression scores (RGS), with effects comparable to or even greater than those observed with progesterone, followed by the intermediate dose (50 mg/kg), albeit with lower magnitude.

Notably, cystic involution was not required for the initial analgesic effects of ambroxol, as evidenced by the Abx 10 group, which showed early reductions in mechanical hyperalgesia despite the absence of significant effects on lesion volume. This finding suggests that the early analgesic response may be mediated by intrinsic anti-inflammatory or neuromodulatory properties of the drug. However, the decline in motor performance observed during the final week in this group indicates that, in the absence of antiproliferative and cyst-involutive effects at this dose, the analgesic effect may not be sustained over time. Conversely, higher doses were associated with more stable and sustained responses.

We propose that the observed analgesic profile may be related to a combination of mechanisms attributed to ambroxol, including its well-established action on voltage-gated Na^+^ channels. Based on the findings of the present study, oral administration of ambroxol hydrochloride demonstrated a favorable pleiotropic profile, particularly at intermediate and higher doses, with progressive consolidation of its effects over time with continuous treatment.

Ambroxol appears to act on multiple pathways involved in the pathogenesis of endometriosis, promoting significant analgesic and anti-inflammatory effects, a substantial reduction in histological scores and inflammatory cytology, as well as marked cystic involution. Collectively, these findings support further investigation of ambroxol as a potential non-hormonal therapeutic option or adjuvant in the management of this condition.

In comparing outcomes across treatment groups, progesterone served as the reference treatment within our protocol, demonstrating consistent effects across all assessed domains over time. No statistically significant differences were observed when compared with higher doses of ambroxol, particularly 100 mg/kg/day, with a similar magnitude of response.

This difference in dosing regimens reflects the distinct pharmacokinetic and pharmacodynamic profiles of the treatments. Medroxyprogesterone acetate administered intramuscularly exhibits a depot-like profile, characterized by an initial rise in systemic concentrations followed by a gradual decline and sustained exposure over time, rather than a rapid loss of activity [23,24,25].

Importantly, the biological effects of progestins are not determined exclusively by peak serum concentrations. Their pharmacodynamic activity is mediated through progesterone receptor signaling, involving genomic regulation of pathways related to inflammation, cell proliferation, and tissue remodeling. Consistently, current reviews and clinical guidelines describe progestins as first-line therapies acting through sustained hormonal modulation rather than immediate peak-dependent effects [2,23].

Mechanistic evidence further supports the genomic and pharmacodynamic basis of progestin activity in endometriosis. Progesterone receptor signaling has been shown to regulate the transcription of genes involved in inflammation, cell proliferation, and tissue remodeling, modulating inflammatory responses and suppressing estrogen-driven proliferation in endometriotic tissue through downstream genomic mechanisms [26].

In parallel, progestins induce decidualization and progressive atrophy of endometrial tissue, with therapeutic effects dependent on sustained receptor activation and cumulative downstream signaling rather than immediate pharmacological action [27]. Together, these mechanisms support a time-dependent pharmacodynamic response, consistent with the progressive therapeutic effects observed in the present study.

This framework supports a time-dependent pharmacodynamic response and helps explain the temporal pattern observed in the present study, in which the progesterone group showed more evident effects at later time points. Such findings are consistent with cumulative biological activity on inflammatory signaling and lesion remodeling, rather than a purely concentration-driven early peak effect.

In the clinical evaluation of pain, progesterone was the only treatment to significantly increase nociceptive thresholds (von Frey test) from the first week, indicating an early and consistent response. This initial effect may be related to the rise in systemic concentrations following intramuscular administration, whereas the more pronounced effects observed at later time points are consistent with prolonged pharmacological activity and the progressive genomic effects of progestins on inflammatory modulation and endometriotic tissue remodeling [2,23,25].

However, it is well recognized that treatment with medroxyprogesterone acetate, in addition to requiring parenteral administration, may not be sustainable in the long term due to adverse effects associated with induced hypoestrogenism, including bone loss, irregular bleeding, weight gain, and cognitive impairment [23,24,28]. In contrast, ambroxol does not directly interfere with ovarian function or its associated physiological processes, which represents a potential therapeutic advantage.

Several limitations should be acknowledged. First, the treatment duration was relatively short (three weeks). In addition, important inflammatory and angiogenic mediators, such as IL-4, IL-17, IFN-γ, TGF-β, TNF-α, NF-κB, and VEGF, were not evaluated. Another limitation was the absence of serial measurements of the cytokines analyzed. Furthermore, oxidative stress markers, including MDA, Nrf2/HO-1, GSH, and SOD, were not assessed, and differential cell counts in the peritoneal lavage were not performed.

Although the a priori sample size calculation indicated a requirement of 48 animals, the final analyzed sample comprised 38 animals due to anesthetic mortality and exclusion of animals that did not develop cystic lesions according to predefined inclusion criteria. These losses are inherent to this type of experimental model and occurred before treatment allocation.

In repeated-measures designs, sample size estimation is sensitive to assumptions regarding the correlation among repeated observations, and plausible variation in this parameter may substantially influence the required sample size. Within a reasonable correlation range of 0.5–0.6, the final sample remained acceptable for evaluation of the primary outcomes, as post hoc power analysis for the final sample of 38 animals indicated statistical power ranging from 0.73 to 0.85. Nevertheless, the reduced sample size may have limited statistical sensitivity to detect subtle differences in secondary outcomes or dose–response relationships.

Therefore, future investigations in experimental endometriosis should adopt expanded methodological approaches. These include longer treatment durations and diversified dosing regimens, such as neoadjuvant use or combination with other agents. In addition, stepped dosing or dose-tapering strategies should be evaluated to determine therapeutic equivalence and potential inclusion in pharmacological rotation protocols. Finally, future studies should incorporate a more comprehensive panel of cytokines and oxidative stress markers.

## 4. Materials and Methods

### 4.1. Study Characterization

This randomized preclinical experimental study was conducted at the Experimental Laboratory for the Study of Pain (LEED), located at the Don Delgado University Campus of the Federal University of Maranhão (UFMA), between August and September 2024.

### 4.2. Ethical Aspects

The study was approved by the Ethics Committee on Animal Use (CEUA-UFMA) under protocol number SEI 23115.034119/2023-99.

### 4.3. Study Population

Sample size was determined a priori using statistical power analysis performed with G*Power software (version 3.1.9.7; Heinrich Heine Universität Düsseldorf, Germany), based on a repeated-measures ANOVA design with intra- and intergroup interactions. The initial calculation indicated a requirement of 48 animals (8 per group) to detect biologically relevant differences, with a 95% confidence level and a significance level of 5% (α = 0.05). Statistical power (1 − β) was set at 80% (β = 0.20), corresponding to a maximum 20% probability of a type II error. An effect size (f) of 0.25 was assumed.

The study included adult female Wistar rats (*Rattus norvegicus albinus*), aged two months, nulliparous, weighing 200–300 g, obtained from the Central Animal Facility, UFMA.

### 4.4. Experimental Anesthetic Procedure

Following a 12 h fast, the rats were weighed and anesthetized via intramuscular injection (posterior aspect of the right thigh) of ketamine (75 mg/kg) and xylazine (10 mg/kg). Anesthetic maintenance was performed using a DL700 Lite inhalation anesthesia system (DeltaLife^®^, São José dos Campos, SP, Brazil) with isoflurane at 1.5 Minimum Alveolar Concentration (MAC), ensuring adequate hypnosis, immobility, and muscle relaxation throughout the procedure. Vital signs were continuously monitored. Anesthetic depth was continuously monitored by evaluating the corneal and tail-flick reflexes, and oxygen supplementation was provided during inhalational anesthesia.

During anesthetic induction for the surgical procedure, four animals died unexpectedly and were excluded prior to further experimental procedures. Random allocation to experimental groups was performed only after surgical induction of endometriosis and subsequent ultrasonographic confirmation of endometriotic cyst implantation. The final number of animals included in each group after all exclusions is described in Section 4.7.

### 4.5. Surgical Induction of Experimental Endometriosis

Once anesthetized, the animals were placed in the supine position on the surgical table, with the limbs secured in abduction, and were maintained under continuous monitoring of vital signs (heart and respiratory rate) using a multiparameter monitor (ECG-DL650, DeltaLife^®^, São José dos Campos, SP, Brazil). The operative field in the caudoventral abdominal region (4 cm × 5 cm) underwent manual hair removal, followed by mechanical cleansing and antisepsis with a 10% povidone–iodine solution (Riodeine^®^, Rioquímica, São José do Rio Preto, SP, Brazil), and placement of a fenestrated sterile drape.

Autotransplantation technique was performed as follows: a 3 cm midline incision was made using a No. 15 scalpel blade, involving the skin, musculoaponeurotic layer, and peritoneum, followed by identification of intracavitary organs and exposure of the uterus, adnexa, and mesentery. Subsequently, the middle third of the left uterine horn was identified, grasped, ligated, and transected using 6-0 nylon suture (Mononylon^®^, Ethicon, Somerville, NJ, USA) with three simple sutures to ensure hemostasis [29,30].

Concomitantly, the uterine specimen was sectioned into longitudinal slices followed by transverse cuts to obtain a 4.5 mm × 4.5 mm flap, which was maintained in Ringer’s lactate solution (Ringer Lactato^®^, B. Braun, Melsungen, Germany). A transfixing suture using 6-0 nylon thread was placed on the serosal surface of the flap, which was then kept tagged.

**Figure 8:**
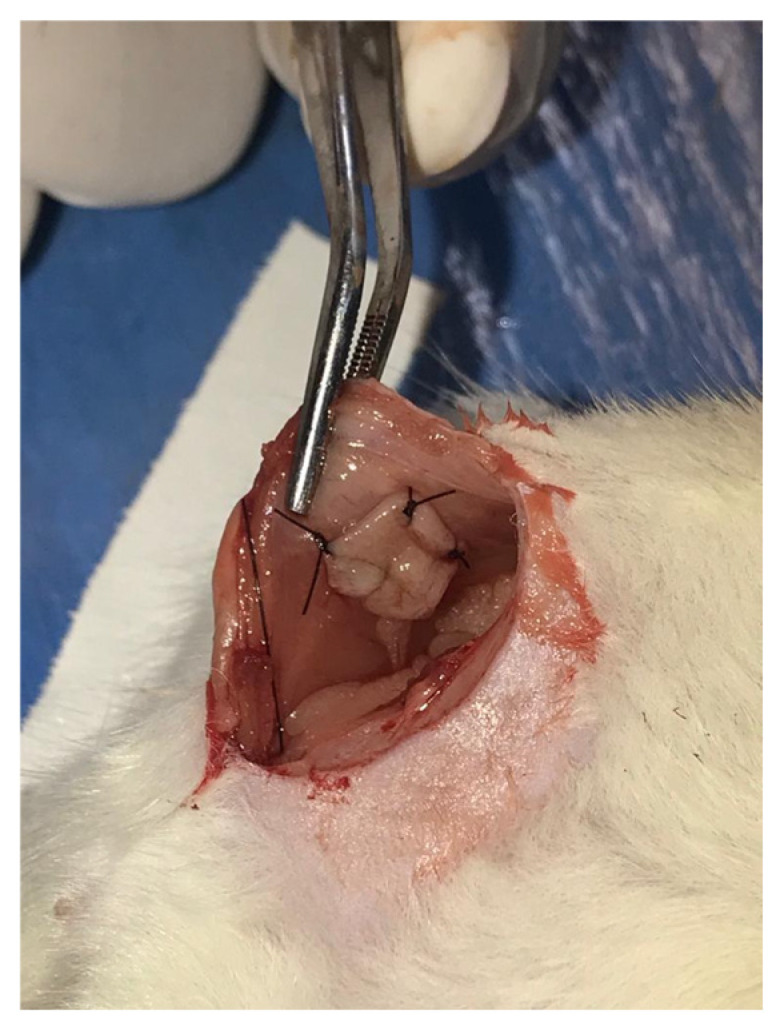
Uterine autotransplant fragment fixed to the right parietal peritoneum of a Wistar rat.

The flaps were autotransplanted onto the right parietal peritoneal surface of the abdominal wall, maintaining the serosal surface in contact with the parietal peritoneum and the endometrial surface facing the peritoneal cavity. Fixation was achieved using simple sutures on the previously tagged flap (Figure 8). Throughout the procedure, the peritoneal cavity was covered with gauze soaked in 0.9% sodium chloride solution. Abdominal wall closure was performed in two planes using continuous sutures involving the musculoaponeurotic layer and the skin, with 5-0 nylon.

Immediate post-anesthetic recovery occurred in a controlled environment, and no hormonal supplementation was administered before or after laparotomy. After the surgery, all animals were observed for 21 days in the animal facility without additional pharmacological interventions, except for immediate postoperative analgesia consisting of dipyrone (100 mg/kg) + morphine (2.5 mg/kg), administered as a single subcutaneous dose. Additional analgesic doses were available on demand during the postoperative period [31].

### 4.6. Ultrasonographic Confirmation of Experimental Endometriosis and Inclusion Criteria

Twenty-one days after the implantation, the rats were again subjected to inhalational anesthesia with isoflurane at 1.5 MAC to identify and measure the dimensions of the autotransplanted foci. A portable ultrasound device (LOGIQ E^®^, GE HealthCare, Chicago, IL, USA) equipped with a high-frequency linear transducer (14–22 MHz) was used. All examinations were performed by a single experienced ultrasonographer who was blinded to the experimental groups.

Implant growth was classified according to the Quereda classification (Table 3) [32]. Only implants classified as grade II or III cystic growth, defined as those with the largest diameter measuring 2.0 mm or greater, were included in the study.

After the surgical induction period, ultrasonography revealed that six animals did not develop cystic lesions meeting the predefined inclusion criteria and were therefore euthanized (as described in Section 4.11) and excluded from the study. Consequently, the final analyzed sample consisted of 38 animals distributed across the experimental groups. No additional exclusions occurred after treatment initiation.

In addition to measuring implant diameters, ultrasonography also provided volumetric measurements. These values enabled comparison of implant volumes before and after treatment, which were assessed using the same ultrasound device by the same ultrasonographer.

### 4.7. Treatment Protocol

After ultrasonographic confirmation, the rats were randomly allocated to six groups, and treatment protocols were initiated according to group assignment: Control(−) (*n* = 6), which received oral gavage with 0.9% sodium chloride solution (0.5 mL/100 g body weight) for 21 days [30]; Progesterone (*n* = 6), which received a single intramuscular dose of medroxyprogesterone acetate (15 mg/kg) on the first day of treatment [33,34,35]; Sham (*n* = 5), which did not undergo endometriosis induction and received no treatment; and three ambroxol-treated groups receiving daily oral gavage for 21 days: Abx 10 (10 mg/kg; *n* = 7), Abx 50 (50 mg/kg; *n* = 7), and Abx 100 (100 mg/kg; *n* = 7).

Doses were determined using allometric scaling based on body surface area to appropriate translation from human clinical doses (ranging from standard respiratory doses to high-dose regimens used in acute respiratory distress syndrome (ARDS)) to the rat model [34,36]. A standard human body weight of 60 kg was assumed for the dose conversion, in accordance with established interspecies scaling principles.

Considering that ambroxol doses used in human clinical practice range from 20 to 75 mg/day for the treatment of mild respiratory diseases to much higher doses of 1–3 g/day used in ARDS management [37,38], application of the dose conversion formula yields approximate equivalents of 2.04–7.71 mg/kg and 102.8–308.3 mg/kg, respectively, in the rat model.

Accordingly, doses of 10, 50, and 100 mg/kg of ambroxol were selected to encompass the main therapeutic ranges used in these clinical contexts. The highest dose tested (100 mg/kg) was chosen because it lies within the upper range of the human-to-animal allometric scaling while remaining below the maximum doses previously reported as safe and effective in experimental pain and inflammatory models, thereby allowing evaluation of potential dose-dependent effects without exceeding pharmacologically relevant exposure levels.

### 4.8. Blinding

All outcome assessors responsible for behavioral testing, biochemical assays, and histological analyses were blinded to group allocation throughout data collection and analysis. Treatments were administered by a separate researcher who was not involved in outcome assessment. Data analysis was performed using coded datasets to maintain blinding until completion of the statistical evaluation.

### 4.9. Clinical Assessment of Pain

Behavioral assessment of pain was initiated 24 h after ultrasonographic confirmation of ectopic endometrial implantation. Evaluations were performed before treatment initiation (Day 0, D0) and on D7, D14, and D21.

Pain assessments were conducted by direct observation. Prior to the evaluations, animals underwent a 7-day habituation period to minimize stress-related interference and ensure consistency in behavioral responses.

To minimize bias related to false-positive findings in behavioral tests due to routine handling (e.g., cage cleaning, drug administration, and animal manipulation) and environmental factors (temperature, noise, and lighting), all nociceptive and spontaneous pain assessments were performed before gavage. This approach ensured that handling associated with treatment administration did not interfere with the evaluations. Additionally, room temperature and lighting conditions were maintained similar to those of the animal facility, and testing was conducted in a quiet environment, with only the two evaluators present in the room [39].

#### 4.9.1. Assessment of Spontaneous Pain

Spontaneous pain was assessed using the Rat Grimace Scale (RGS), a validated behavioral tool based on facial expression analysis in rodents [40,41,42]. This method does not require prior training and can be performed in the home cage, minimizing environmental interference.

Facial action units evaluated included orbital tightening, nose/cheek flattening, ear changes, and whisker position [43,44]. Each parameter was scored as follows: 0 (absence of pain), 1 (moderate pain), and 2 (marked pain). The overall RGS score was calculated as the mean of the scores assigned by three blinded evaluators [45].

#### 4.9.2. Assessment of Mechanical Allodynia

Mechanical allodynia was assessed using the von Frey test with an electronic von Frey-type aesthesiometer (Insight^®^, Insight Equipamentos Científicos, Ribeirão Preto, SP, Brazil), consisting of a pressure transducer connected to a digital force counter expressed in grams (g), as previously described for mechanical nociceptive threshold assessment [46,47]. A disposable polypropylene tip (0.5 mm diameter) was used to apply stimuli to the anterior pelvic region.

Animals were individually placed in transparent acrylic boxes measuring (12 cm × 20 cm × 17 cm) on a wire mesh platform (5 mm^2^ grid) and allowed to habituate for 15 min prior to testing. A mirror positioned 25 cm below the platform facilitated visualization of the pelvic region.

A single blinded evaluator applied a linearly increasing force through the mesh grid to the pelvic region until an abdominal withdrawal response defined as an abdominal defensive movement (retraction/withdrawal). Stimuli were repeated up to six times until three consistent withdrawal responses were obtained. The nociceptive threshold was defined as the mean of these three measurements. Intervals of approximately 60 s were allowed between stimuli to minimize sensitization. Nonspecific behaviors (e.g., grooming or exploratory activity) were not considered valid responses.

This approach represents an adaptation of von Frey-based mechanical stimulation for assessment of abdominal sensitivity in visceral pain models, as described in previous studies of visceral hypersensitivity [48,49].

#### 4.9.3. Assessment of Motor Activity/Forced Ambulation

Motor performance was assessed using a rotarod apparatus (IITC Life Science, Woodland Hills, CA, USA) operating at a constant speed of 16 revolutions per minute for 300 s.

The quality of forced ambulation was graded using an adapted numerical scale ranging from 5 to 1, as follows: 5, normal limb use; 4, mild claudication; 3, severe claudication; 2, intermittent limb disuse; and 1, complete limb disuse [50].

### 4.10. Blood Collection

On D21, prior to euthanasia, animals were anesthetized with isoflurane at 1.5 MAC. Whole blood was then collected by aortic puncture into EDTA-containing tubes to preserve cellular and plasma components.

#### 4.10.1. Plasma Separation

Blood samples were centrifuged at 3000× *g* for 15 min to separate cellular components from plasma. The plasma fraction was carefully transferred to microtubes (Eppendorf^®^, Hamburg, Germany) and stored at −80 °C until analysis.

#### 4.10.2. Cytokine Quantification

IL-1β levels were quantified using an enzyme-linked immunosorbent assay (ELISA), in accordance with the manufacturers’ instructions (ELK Biotechnology^®^, Wuhan, China and R&D Systems, Minneapolis, MN, USA). Absorbance was measured using an ELISA microplate reader (KASUAKI^®^, model DR-200Bc; DIATEK Instruments, Wuxi, China). All measurements were performed in triplicate for each sample to ensure analytical reliability and reproducibility.

### 4.11. Peritoneal Lavage and Leukocyte Counting

On D21, peritoneal lavage was performed under aseptic conditions. A volume of 10 mL of phosphate-buffered saline (PBS, pH 7.4), previously cooled to 4 °C, was injected into the peritoneal cavity of each animal [51,52]. This was followed by gentle abdominal massage for 1 min to facilitate cell detachment, after which the fluid was carefully aspirated using a sterile syringe and immediately transferred to microtubes kept on ice [53].

Samples were centrifuged at 400× *g* for 10 min at 4 °C. The resulting cell pellet was resuspended in red blood cell lysis buffer [54]. After incubation for 5 min at room temperature, samples were centrifuged again at 400× *g* for 10 min, and the pellet was resuspended in sterile PBS.

For cell quantification, 10 µL aliquots were loaded into a Neubauer chamber and analyzed under light microscopy [52]. Total leukocyte counts (cells/mL) were calculated by multiplying the mean number of cells per large square by the chamber correction factor (10^4^) and the dilution factor introduced during the lysis procedure [51,53].

### 4.12. Euthanasia

On D21, animals in each group were euthanized by intramuscular injection of an anesthetic solution containing ketamine hydrochloride (300 mg/kg) and xylazine hydrochloride (30 mg/kg), in accordance with Resolution No. 1000 of 11 May 2012, of the Federal Council of Veterinary Medicine [55]. Death was confirmed by respiratory arrest and absence of reflexes [56].

After inspection of the abdominal cavity, endometrial implantation sites were identified and carefully excised. The specimens were rinsed with 0.9% sodium chloride solution and placed in labeled containers containing 10% buffered formalin.

### 4.13. Histological Analysis of Ectopic Endometrial Tissue

Histological analysis was performed at the Multiuser Histology Laboratory of the Graduate Program in Health Sciences, UFMA. Endometriotic implant samples from the abdominal wall of animals in the Control(−), treated, and Sham groups were collected on D21 and immediately fixed in 10% buffered formaldehyde (pH 7.2). After 48 h, samples underwent standard histological processing, including dehydration in graded ethanol, clearing in xylene, and paraffin embedding [57,58].

Tissue sections (3–5 µm) were obtained extending from one edge of the implant to the other and from the cystic epithelial surface to the underlying muscle layer, and were stained with H&E. Images were acquired at different magnifications using a camera coupled to an ICC50 light microscope (Leica Microsystems^®^, Wetzlar, Germany) equipped with 10× eyepieces and 4× (500 µm), 10× (200 µm), and 40× (50 µm) objectives. Histological evaluation was performed by direct observation by a single trained examiner blinded to group allocation.

Persistence of epithelial cells in the autotransplants after treatment was evaluated semi-quantitatively using a histological scoring system (Table 4) [18], in which greater impairment of epithelial layer integrity was interpreted as indicative of greater regression of endometriotic implants.

### 4.14. Statistical Analysis

Data distribution was assessed for normality using the Shapiro–Wilk test, and homogeneity of variances was evaluated using Levene’s test. As the assumptions for parametric analysis were met, parametric methods were used for group comparisons.

Comparisons among experimental groups were performed using one-way analysis of variance (one-way ANOVA) or, when two sources of variability were present, two-way analysis of variance (two-way ANOVA). When appropriate, Tukey’s post hoc test was applied for multiple comparisons.

Data were analyzed using GraphPad Prism^®^ software (version 7.0; GraphPad Software, San Diego, CA, USA). A *p*-value < 0.05 was considered statistically significant.

## 5. Conclusions

Oral administration of ambroxol (10, 50, and 100 mg/kg/day) in an experimental model of endometriosis resulted in the following outcomes:

- Antinociceptive activity: Significant increases in nociceptive threshold and reductions in both primary mechanical hyperalgesia and spontaneous pain.
- Volumetric reduction: Doses of 50 and 100 mg/kg significantly reduced endometriotic implant volume, with effects comparable to medroxyprogesterone acetate.
- Anti-inflammatory activity: Marked reductions in IL-1β levels were observed, with efficacy comparable to progesterone.
- Epithelial disruption: Ambroxol significantly disrupted the cystic epithelial lining of endometriotic implants, contributing to lesion regression, similarly to progesterone.

These findings indicate that ambroxol exerts a multimodal effect, targeting both sensory and structural components of endometriosis, and support its potential as a repurposed non-hormonal therapeutic strategy for disease management.

## Figures and Tables

**Table 1:** Variation in the size of endometriotic foci after 21 days of treatment with oral 0.9% saline solution (1.0 mL/day), oral ambroxol (10, 50, and 100 mg/kg/day), or a single intramuscular dose of medroxyprogesterone (15 mg/kg).

	Initial Volume (mm^3^) ± SD	Final Volume (mm^3^) ± SD	Absolute Var. (mm^3^) ± SD (Var. %)	*p* *
Control(−) (A)	47.85 ± 9.94	40 ± 5.77	7.85 ± 4.17 (16.4%)	0.8225
Abx 10 (B)	54.28 ± 7.86	38.57 ± 6.90	15.71 ± 0.96 (28.9%)	0.0797
Abx 50 (C)	41.42 ± 8.99	21.42 ± 6.90	20 ± 2.09 (48.2%)	0.0111
Abx 100 (D)	45.71 ± 9.75	20 ± 16.03	25.71 ± 6.28 (56.2%)	0.0006
Progesterone (E)	45.71 ± 12.72	14.28 ± 5.34	31.43 ± 7.38 (68.7%)	<0.0001
*p* **	0.9360 (A vs. B)	>0.9999 (A vs. B)		
	0.9360 (A vs. C)	0.0220 (A vs. C)		
	>0.9999 (A vs. D)	0.0111(A vs. D)		
	>0.9999 (A vs. E)	0.0006 (A vs. E)		
	0.2435 (B vs. C)	0.0425 (B vs. C)		
	0.7434 (B vs. D)	0.0220 (B vs. D)		
	0.7434 (B vs. E)	0.0013 (B vs. E)		
	0.9955 (C vs. D)	>0.9999 (C vs. D)		
	0.9955 (C vs. E)	0.8875 (C vs. E)		
	>0.9999 (D vs. E)	0.9681 (D vs. E)		

* Two-way ANOVA followed by Tukey’s multiple comparisons test was used to compare initial and final volumes within each group (D0 vs. D21). ** The same analysis was used to compare mean volumes between groups at each time point (D0 and D21). *p* < 0.05 was considered statistically significant.

**Table 2:** Comparison of semi-quantitative epithelial persistence scores among groups treated with oral 0.9% saline solution (0.5 mL/day), oral ambroxol (10, 50, and 100 mg/kg/day), or a single intramuscular dose of medroxyprogesterone (15 mg/kg).

	Mean ± SD
Control(−) (A)	2.83 ± 0.40
Abx 10 (B)	2.57 ± 0.53
Abx 50 (C)	1.57 ± 0.78
Abx 100 (D)	1.28 ± 0.75
Progesterone (E)	1.16 ± 0.98
*p* *	0.9885 (A vs. B)
	0.0366 (A vs. C)
	0.0064 (A vs. D)
	0.0033 (A vs. E)
	0.1013 (B vs. C)
	0.0182 (B vs. D)
	0.0116 (B vs. E)
	0.9990 (C vs. D)
	0.8703 (C vs. E)
	0.9990 (D vs. E)

* Two-way ANOVA followed by Tukey’s multiple comparisons test was used to compare mean epithelial persistence scores between groups. *p* < 0.05 was considered statistically significant.

**Table 3:** Classification of endometrial implant growth (adapted from [32]).

Growth Grade	Implant Characteristics
0	The implant disappears or, if visible, does not develop into a cyst.
I	The implant forms a vesicle whose largest diameter is <2 mm or, if larger, is solid
II	The implant forms a fluid-filled cyst, with the largest diameter ≥ 2 mm but <4.5 mm (smaller than the initial implant size).
III	The vesicle diameter is similar to or greater than the initial implant size (≥4.5 mm).

**Table 4:** Semi-quantitative scoring system for evaluating epithelial cell persistence in endometrial autotransplants (adapted from [18]).

Score	Epithelial Characteristic
3	Well-preserved epithelial layer
2	Moderately preserved epithelium with leukocytic infiltrate
1	Poorly preserved epithelium, with only occasional epithelial cells
0	Absence of the epithelial layer

## Data Availability

The original contributions presented in the study are included in the article, further inquiries can be directed to the corresponding authors.
